# Anxiety as a Risk Factor for Cardiovascular Diseases

**DOI:** 10.3389/fpsyt.2016.00025

**Published:** 2016-02-24

**Authors:** Silvia Raquel Soares Ouakinin

**Affiliations:** ^1^University Clinic of Psychiatry and Medical Psychology, Faculty of Medicine, University of Lisbon, Lisbon, Portugal

**Keywords:** anxiety, cardiovascular risk factors, heart rate variability, cardiovascular diseases, emotion regulation

Potential psychological risk factors for cardiovascular diseases (CVD) can be grouped in three domains. The first consists of negative affective states including depression, anxiety, distress, and anger, the second includes personality patterns such as Type A behavior pattern and Type D personality, and the third comprehends social factors including socioeconomic status and social support (1). Anxiety, a significant risk factor for CVD, is recently recognized as a crucial issue in younger and older adults, with a causal relation to other risk factors, such as depression, substance use, overweight, sleep difficulties, or a sedentary life style (2, 3).

Anxiety disorders, with a 12-month prevalence rate of 17.7% and a higher lifetime prevalence in women (30.5%) than men (19.2%), are among the most common psychiatric diagnoses. (4). Several anxiety disorders are developed during childhood and tend to persist if not adequately treated. They include social anxiety disorder, panic disorder, agoraphobia, and generalized anxiety disorder, among others (5).

The association between anxiety and cardiovascular adverse events in patients with CVD is still not clear (6–12); however, recent reviews provide evidences according to which in coronary heart disease, anxiety can increase the risk of major cardiac events and mortality (1, 2, 13).

The physiological pathways that could explain the impact of psychosocial risk factors on the cardiovascular system are not always evident; therefore, this commentary highlights the findings of an association between anxiety disorders and reduced heart rate variability (HRV) by Chalmers et al. (14), aiming to contribute for explaining the comorbidity between anxiety and CVD. HRV represents an indicator of the complex relation between sympathetic and parasympathetic branches of the autonomic nervous system (ANS) and its influence on heart functioning. Low HRV can be conceptualized both as a precursor of disease and a risk factor, with adverse effects on cardiovascular health (15).

In “*Anxiety Disorders are Associated with Reduced Heart Rate Variability: A Meta-Analysis*,” Chalmers et al. (14) described a meta-analysis of 36 studies in order to examine “*the impact of anxiety on resting HRV a psychophysiological marker of health and well-being*.” They rely on the neurovisceral integration model to explain the relation between HRV and anxiety, presenting mixed findings to support this assumption. For clarification, they point to the advantages of a meta-analytic procedure. Their results highlight that “*anxiety disorders are associated with significant reductions in HRV*,” with a “*small-to-moderate effect size*.” Authors discuss those findings in the context of the neurovisceral integration model and the polyvagal theory, pointing to the relevance of the vagal function decrease in the pathophysiology of a state of allostatic load, in response to a threatening environment. Addressing some limitations of this meta-analytic review, they indicate the reduced number of studies in different anxiety disorders and the importance of depression and anxiety comorbidity. In the summary, they refer the implications for patient’s health in a long-term perspective and the need for considering preventive strategies (14).

Despite the differences between anxiety disorders, anxious individuals, in general, overestimate the danger of perceived fearful situations, and this cognitive appraisal is associated with a state of arousal and autonomic activation, an adaptive response to prepare the organism to a “fight or flight” reaction (16). However, the nature of the appraised threats does not always imply such a response. Then, the adaptive response turns maladaptive and potentially toxic. The generalization of this pattern is linked to the concept of anxiety and increased risk for CVD, diabetes, and inflammatory disorders.

Also, evidences for the association between psychological factors, such as depression, hostility, or anger, and the metabolic syndrome, as a risk cluster for those diseases, strengthen the need to address these relations (17).

Evidences in literature emphasize that brain regions responsible for processing threatening information can trigger biological stress-response systems, such as the hypothalamic–pituitary–adrenal (HPA) axis and the ANS. Associated to threat-related brain activity, the sympathetic nervous branch of the ANS is upregulated with an increase in blood pressure and greater catecholamine release from adrenal medulla and sympathetic nerves (norepinephrine). On the contrary, the parasympathetic division is downregulated as shown by decreases in respiratory sinus arrhythmia. Activation of the central and peripheral systems in response to acute social-evaluative threat also generates increased systemic inflammation (16, 18).

Hyperactivity in autonomic response patterns can increase the expression of anxiety symptoms, the interoception of body signals, and central-periphery interactions (19).

Neuroimaging data support the hypothesis of what Eccles et al. called a neurovisceral phenotype model, associating brain regions (the amygdala, cingulate and insula cortex, basal ganglia, and ventromedial prefrontal cortex), subjective feelings, and patterns of affective reactivity in different clinical entities (20). According to Thayer, HRV may display the flexibility of a balanced physiologic regulation or an index of healthy cardiac function, thus offering a sign of degree of adaptive regulation of the “brain’s integrative system” and the organism adaptive skills facing complex interactions with the external milieu (21).

Emotion regulation strategies have been extensively researched as risk factors for the onset of coronary heart diseases. In a Swedish National Study including 46,393 men, in a follow-up of 38 years, Potijk et al. found that in the presence of a parental history of heart disease, poor emotion control in young adults (18–20 years) was a predictive factor of long-term risk, even controlling for lifestyle and biomedical risk factors (22). Interestingly, greater difficulties in emotion regulation have been linked to lower resting vagal-mediated HRV (23), reinforcing their role in the association between major or minor stressors, negative affect, and heart reactivity. The finding that cardiovascular risk markers are related to parasympathetic activity, in adolescents aged 11–14 years, suggests that HRV could be a potential marker for discriminating adolescents at risk (24).

The implications of poor emotion regulation strategies, higher levels of anxiety or depression, and psychophysiological dysregulation seem to be the most relevant in prevention and treatment, with significant impact in morbidity and mortality in anxious as well as in CVD patients. Additional important issues, such as gender-related differences in cardiovascular risk and early determinants of autonomic response in adulthood, deserve further investigation.

## Author Contributions

The author confirms being the sole contributor of this work and approved it for publication.

## Conflict of Interest Statement

The author declares that the research was conducted in the absence of any commercial or financial relationships that could be construed as a potential conflict of interest.
